# Peroxisome Proliferator-Activated Receptor-**γ**-Mediated Polarization of Macrophages in *Leishmania* Infection

**DOI:** 10.1155/2012/796235

**Published:** 2012-02-01

**Authors:** Marion M. Chan, Nagasuresh Adapala, Cui Chen

**Affiliations:** ^1^Department of Microbiology and Immunology, Temple University School of Medicine, Room 534 OMS, 3400 North Broad Street, Philadelphia, PA 19140, USA; ^2^Department of Anatomy and Cell Biology, Temple University School of Medicine, Philadelphia, PA 19140, USA; ^3^Undergraduate Research Program, College of Science and Technology, Temple University, Philadelphia, PA 19140, USA

## Abstract

Infection is the outcome of a contest between a pathogen and its host. In the disease leishmaniasis, the causative protozoan parasites are harbored inside the macrophages. *Leishmania* species adapt strategies to make the infection chronic, keeping a balance between their own and the host's defense so as to establish an environment that is favorable for survival and propagation. Activation of peroxisome proliferator-activated receptor (PPAR) is one of the tactics used. This ligand-activated nuclear factor curbs inflammation to protect the host from excessive injuries by setting a limit to its destructive force. In this paper, we report the interaction of host PPARs and the pathogen for visceral leishmaniasis, *Leishmania donovani*, *in vivo* and *in vitro*. PPAR expression is induced by parasitic infection. Leishmanial activation of PPAR**γ** promotes survival, whereas blockade of PPAR**γ** facilitates removal of the parasite. Thus, *Leishmania* parasites harness PPAR**γ** to increase infectivity.

## 1. Leishmaniasis

Leishmaniasis is caused by parasitic protozoa of the genus *Leishmania*. The disease is found worldwide, with an estimated prevalence of 12 million cases, 50,000 annual deaths, and 350 millions of the world's population at risk [1]. *Leishmania* has two stages in its life cycle: flagellated promastigotes that live within the alimentary canal of the insect vector and amastigotes that multiply within the phagolysosomes of mammalian macrophages. Infected female sandflies introduce saliva and promastigotes into the mammalian host during blood meals. The promastigotes are taken by leukocytes and differentiate into intracellular amastigotes within the macrophages. Then, infected macrophages carry the parasites to different organs. Over twenty species are known to infect humans. The cutaneous species reside and multiply within the skin tissue, whereas the visceral species predominantly accumulate in the liver, spleen, and bone marrow. These diverse species cause different clinical manifestations, varying from self-healing or metastasizing skin lesions to enlargement of visceral organs including the liver and spleen. The disease symptoms are classified as cutaneous, mucocutaneous, or visceral leishmaniasis.

## 2. Resistance versus Susceptibility

Immunity against all species of *Leishmania* uniformly relies on a type 1 immune response that produces interferon *γ* (IFN*γ*). Produced by T helper 1 cells, IFN*γ* activates macrophages to generate nitric oxide (NO), a free radical that can kill *Leishmania*. Type 2 immune response, on the other hand, is ineffective [2]. Production of interleukin-4 (IL-4) in *Leishmania major* infection, regulatory T cells in *L. mexicana* infection, and IL-10 in infection of various species are all associated with susceptibility [2–6]. It is well established that IL-4 exacerbates leishmaniasis when added exogenously, and IL-10 mutant mice become resistant to infection. However, to date, the reason why this cytokine promotes the pathogenesis of *Leishmania* infection remains partially understood [7–9].

## 3. M1 versus M2 in Disease Pathogenesis

Macrophages, the host of *Leishmania *parasites, are markedly heterogeneous. When stimulated by IFN*γ*, these macrophages differentiate into the classically activated (M1) phenotype, with inducible nitric oxide synthase (iNOS) which produces NO from arginine. Intracellular *Leishmania* parasites are eliminated by this subpopulation. On the contrary, IL-4 differentiates macrophages towards the alternatively activated (M2) phenotype, which promotes humoral immunity and tissue repair. This subpopulation produces IL-10, and transforming growth factor-*β* (TGF-*β*), [10]. 

In terms of signaling, IL-4 induces the expression of PPAR*γ* and PPAR gamma coactivator-1 (PGC-1) *β* protein through the STAT-6 pathway [12]. This nuclear regulator polarizes the monocytes into alternatively activated (M2) macrophages with anti-inflammatory properties. By its transcriptional activity, it mediates the expression of arginase-1 (Arg1) and CD36 [13, 14]. Arginine metabolism away from production of NO compromises the ability of infected macrophage to clear the intracellular pathogens [2, 11]. CD-36 is a scavenger receptor that mediates phagocytosis and facilitates the removal of apoptotic cells. By its transrepressive action, PPAR*γ* blocks the expression iNOS as well as nuclear factor kappa B (NF*κ*B)-mediated transcription of proinflammation mediators [15].

## 4. *Leishmania donovani* Infection Induces Host PPAR Gene Expression *In Vivo* and *In Vitro*

**Figure 1:**
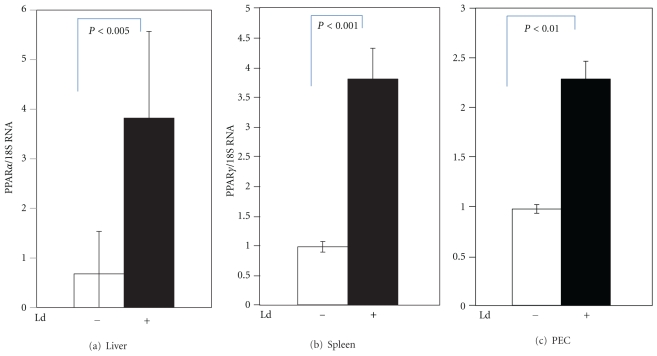
*Leishmania* infection activates PPAR gene expression. In (a) and (b), BALB/c mice were infected i.v. with 10^7^ stationary phase promastigotes of *L. donovani* for 4 weeks, and then liver and spleen, respectively, were harvested. In (c), peritoneal exudate cells (PECs) were obtained from the peritoneum of normal BALB/c mice, infected with* L. donovani* at 1 : 10 ratio, and then harvested for RNA isolation after 2 days. PPAR activation was measured by real-time RT-PCR with normalization to 18S or actin RNA, and modulation was compared and expressed relative to the uninfected control using the delta-delta Ct method. The infected groups (black bars) showed higher levels of gene expression in liver (a), spleen (b), and PECs (c) in comparison to uninfected controls (open bars). Statistical analysis was performed by Mann-Whitney *U* test, and a *P* *value* of less than 0.05 was considered as significant.

**Figure 2:**
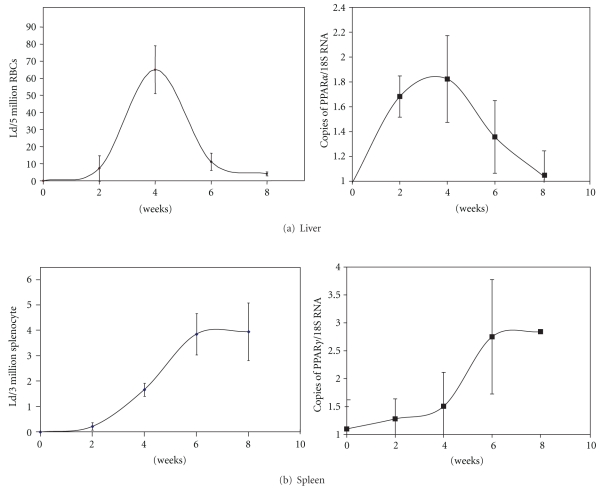
Kinetics of PPAR expression and parasitic infection. BALB/c mice were intravenously injected with 10^7^ stationary phase promastigotes of *L. donovan*i 1S. At two-week intervals, groups (*n* = 5) were sacrificed, and their liver and spleen were harvested. PPAR expression was determined by real-time RT-PCR as described in legend of Figure 1. The amount of parasites in the organs was determined by limiting dilution analysis according to the procedure of Titus and colleagues [17]. Cells were plated into wells of 96-well plates at a range of concentrations, according to the number of red blood cells in the liver and splenocytes in the spleen suspensions and then incubated at 27°C to allow the parasites to transform from intracellular amastigotes into promastigotes. After 2-3 weeks of proliferation, the number of wells that shows parasite growth was scored under a microscope, and the L-Calc software for limiting dilution analysis (provided by Stem Cell Technology, Vancouver, Canada) was used to determine the frequency of parasite. Normalization was based on the number of red blood cells in the liver cultures (a) and the number of splenocytes in the spleen cultures (b).

**Figure 3:**
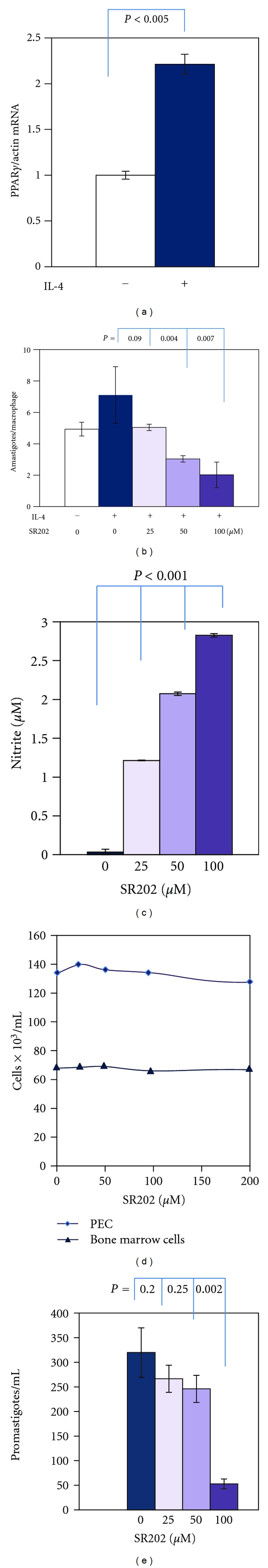
Blocking PPAR*γ* activation with an antagonist reduces* L. donovani *infectivity. In (a), peritoneal exudate cells were infected by *L. donovani* using a 1 to 10 : PEC to promastigote ratio. The cultures were incubated with 4 ng/mL of IL-4 for 24 hours, then total RNA was harvested, and RT-PCRs were performed to quantify the copies of PPAR*γ* and *β*-actin mRNA as described in Adapala and Chan [16]. In (b), PECs from C57/BL6 mice were attached to cover slips and infected with *L. donovani* promastigotes at 1 : 5 ratio. After 20 to 24 hours, 5 ng/mL of IL-4 and various concentration of SR202 were added. The infection was allowed to develop at 37°C in a 5% CO_2_ incubator for another 3 days. Then, the cover slips were fixed in methanol and stained with Diff-Quik. The degree of parasite burden was determined by enumeration under a microscope in a double-blind manner by at least two individuals. Uninfected macrophages, infected macrophages, and the number of amastigotes in these macrophages were counted. The result is reported as amastigotes/macrophage, and each data point was derived from counting at least about one hundred macrophages or one hundred infected macrophages, as appropriate. (c) shows the levels of nitric oxide in PECs that were similarly infected with *L. donovani* promastigotes, except that IFN*γ* was added instead of IL-4 to stimulated inducible nitric oxide synthase expression. On day 5, the amount of nitric oxide was determined with Griess reagent. Shown are the relative levels of nitrite, oxidized form of nitric oxide, in the culture supernatants. In (d), SR202 was added to freshly harvested, uninfected PECs and bone marrow cells at various concentrations. After 3 days, the number of live cells was determined by counting with trypan blue. In (e), SR202 was added to promastigotes, and after 5 days of proliferation, the number of parasites was determined by counting under a microscope. The results shown are representative of three independent experiments.

Our laboratory has been studying the pathogenesis of *Leishmania*, with particular interest in *L. donovani*. We investigated whether susceptibility to infection is associated with the activation of PPAR [16]. Mice of the susceptible BALB/c strain were infected with stationary phase promastigotes of *L. donovani. *After four weeks, their liver and spleen were excised, and PPAR*α* and *γ* mRNA levels were analyzed, using the technique of quantitative real-time RT-PCR. Infection of *Leishmania* leads to increase in PPAR gene expression. We detected 3-fold increase in mRNA of PPAR*α* in the liver (Figure 1(a)) and 3-fold increase for PPAR*γ* in the spleen (Figure 1(b)), as compared to the uninfected control organs. At the cellular level, when residential macrophages from peritoneal exudates of BALB/c mice were infected, PPAR*γ* gene expression was also increased. The increase of PPAR*γ* mRNA was 2-fold for *Leishmania*-infected peritoneal exudate cells (PECs) (Figure 1(c)). Kinetics study was performed to examine whether PPAR expression correlates to parasite burden (Figure 2). The expression of PPAR*γ* in the liver was found to be slightly ahead of the increase in parasites burden (Figure 2(a)). Both PPAR*γ* and parasite number peaked at 4 weeks, which is the time when granuloma will form and parasite growth will be quenched. As the infection in the liver decreases, the expression of PPAR*γ* subsides in coordination. In the spleen where the parasites will persist, the rise in PPAR*γ* expression correlates closely with the increase in parasite number (Figure 2(b)). Both parameters followed a logarithmic increase between weeks 2 to 4 until reaching a plateau at week 6.

## 5. Possible Mechanisms by Which *Leishmania* Induces PPAR Gene Expression

PPAR is a genetic sensor of fatty acids, and its ligands are produced during the course of *Leishmania* infection. Cyclooxygenase-2 (COX-2) is an enzyme that converts arachidonic acid into various bioactive lipids, including prostaglandin (PG) D_2_, PGE_2_, PGF_2_, thromboxane (TX) B_2_, 15d-PGJ_2_, and prostacyclin. Studies in the murine model of *L. donovani *infection have demonstrated that production of these bioactive lipids is enhanced upon infection [18, 19], and studies with* L. amazonensis* have revealed that COX-2 is needed for establishing infection [20]. Blockade of COX with indomethacin inhibits* L. amazonensis* infection of peritoneal macrophages *in vitro* and reduces the size of lesions in susceptible BALB/c mice.

Among the COX-2 products 15d-PGJ_2_ is a potent endogenous ligand for PPAR [21]. Moreover, *in vitro* addition of PGE_2_ increases the number of amastigotes within macrophages [22]. PGE_2_ can activate the generation of lipoxins, a relatively new class of eicosanoids that are also derived from arachidonic acid, but through lipoxygenase or acetylated COX instead [23, 24]. Lipoxin will shut off inflammatory response when bound to its receptor [25]. In *L. major* infection, addition of exogenous lipoxin A_4_ increases infectivity; this effect has been confirmed by receptor inhibition studies [26]. The eicosanoid downregulates inflammation by promoting clearance of apoptotic neutrophils [25]. *Leishmania* parasites (*L. major, L. donovani, L. mexicana*, etc.) are covered with phosphatidylserine (PS), a major surface characteristic of apoptotic cells, and engulfment of apoptotic cells leads to induction of PPAR [27, 28]. For these reasons, the parasitized macrophages would have activated PPAR and are likely to express an alternatively activated (M2) phenotype. Furthermore, PPAR turns on the expression of CD36, and this scavenger receptor would bind to thrombospondin and facilitate phagocytosis of the apoptotic neutrophils [29], the so-called Trojan horses for *Leishmania* parasites at the site of inoculation, reciprocally in a positive feedback manner [30–32]. Ligand activation of PPAR*γ* augments phagocytic capacity of the alternatively activated macrophages.

## 6. Blockade of PPAR Reduces *Leishmania* Infection

**Figure 4:**
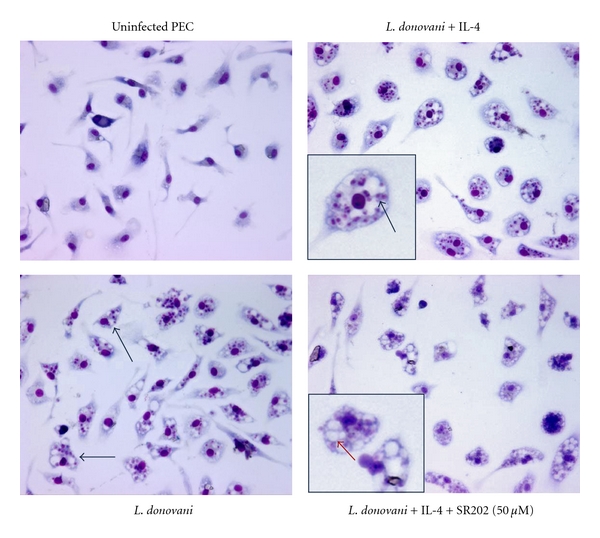
Morphology of the peritoneal macrophages after SR202 treatment. Micrograph of cover slips from the cultures described in Figure 3(b). The black arrows point to macrophages with phagolysosomes filled with amastigotes. The black arrows point to infected macrophages and the red arrow points to infected macrophages with phagolysosomes cleared of amastigotes.

Since PPAR is upregulated with *Leishmania *infection, we proceeded to determine whether the activation of PPAR is essential for infection. Studies assessing the effect of PPAR blockade on *Leishmania* infectivity have been conducted with *L. major*. Our laboratory studied *L. donovani* using PECs from the C57BL/6 mice, a strain that is susceptible to *L. donovani *infection though not *L. major*. A reason for selecting this strain is that it does not have a deficiency in T helper 1 cells and thus is capable of producing IFN*γ*, which is necessary for generation of the parasiticidal NO molecule. Nonelicited residential macrophages from the peritoneum were infected with *L. donovani* promastigotes, and IL-4 was added to activate PPAR*γ* [33]. Then, PPAR*γ* transcriptional activity was blocked with SR202, an antagonist which efficacy and specificity have been shown in adipocytes [34–36]. The effect of PPAR*γ* on parasite survival and proliferation in the host macrophages was assessed by enumerating the number of amastigotes per macrophage. In the absence of IL-4, the number of amastigotes per infected macrophage was 4.94 ± 0.44 (Figure 3(b)). IL-4 activated PPAR*γ* (Figure 3(a)), and this resulted in an increase in infectivity (Figure 3(b)). There were 7.10 ± 1.82 amastigotes per macrophage. This enhancement by IL-4 was reversed by blocking PPAR*γ* (Figure 3(b)). Addition of SR202, at 25 and 50 *μ*M, in a dose-dependent manner, reduced the number of amastigotes per macrophage to 5.05 ± 1.38 and 2.02 ± 0.81, respectively. This reduced infection was further correlated to an increase in nitric oxide level in the cultures (Figure 3(c)). The effect of SR202 at 50 *μ*M or lower is specific to the infectious process for, at these concentrations, the compound did not affect the survival of promastigotes or mammalian cells (Figures 3(d) and 3(e)).  Figure 4 shows the cultures that had been stained with Diff-Quik for microscopic enumeration. The macrophages that had been treated with 50 *μ*M of SR202 presented a healthy morphology with many empty parasitophorous vacuoles freed of parasites.

Complementary results were obtained with *Leishmania major* in bone marrow-derived macrophages from the resistant C57BL/6 mice in a study by Gallardo-Soler et al. (2008). The PPAR antagonists, GW9662 and GW5393, reduce infectivity [13]. Whereas we correlated PPAR activity in our *L. donovani* infection to the transrepressive action on iNOS-mediated NO production (Figure 3), this study correlated infectivity to the transcription of arginase. The enzyme is a bona fide marker for PPAR-mediated transcription and alternatively activated (M2) macrophages. Its mRNA level was decreased in coordination to the decrease in infection. In addition to pharmacological inhibitors, Odegaard et al. (2007) have examined *L. major* infectivity in mice with macrophages that do not express PPAR*γ* (Mac-PPAR*γ* KO, PPAR*γ* ^fl/fl^  LysM^cre^) [37]. The mutant mice have impaired M2 macrophage activation, a delayed disease progression, and a lower parasite load (less footpad swelling) compared to the wild type [37]. Henceforth, PPAR*γ* plays an essential role in the pathogenic process of both *L. major* and *L. donovani. *In the absence of PPAR*γ* activity, the balance shifts from the arginase producing M2 phenotype to that of nitric oxide producing, type 1, response.

## 7. PPAR Activation Enhances *Leishmania* Infection

Conversely, Gallardo-Soler et al. (2008) also demonstrated that the PPAR agonists GW1929 and GW7845 (for PPAR*α*) and GW0742 (for PPAR*γ*), at 1 *μ*m concentration, increased intracellular growth of *Leishmania major *in bone marrow-derived macrophages [13]. When both PPAR/RXR ligands were coadministered, the degree of infection was similar to those infected in the presence of IL-4. This increased number of intracellular amastigotes can be correlated to the levels of arginase activity.

**Figure 5:**
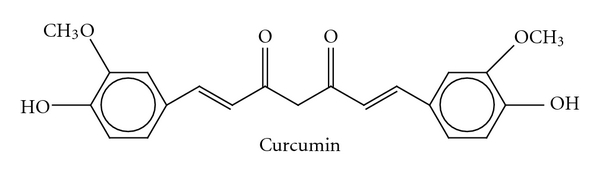
Chemical structure of curcumin.

**Figure 6:**
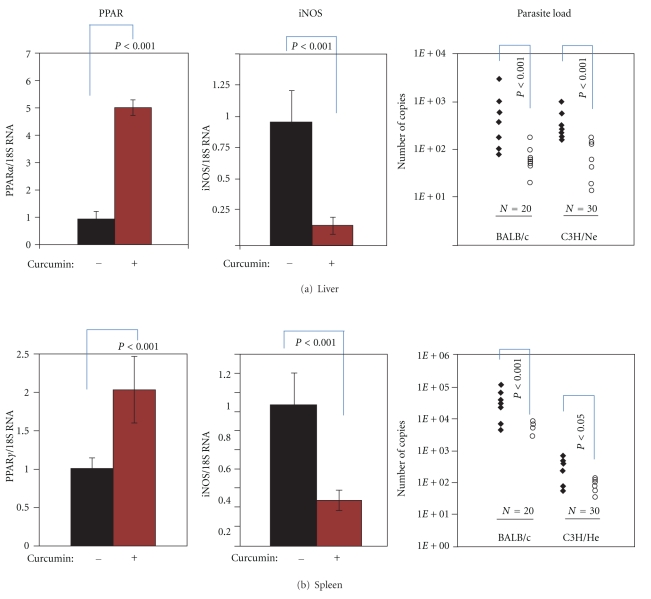
Activation of PPAR enhances parasitic infection. Mice were infected with *L. donovani.* One group was fed with 0.2 mL freshly prepared curcumin solution, given by oral gavage every other day throughout the course of the study. Curcumin was dissolved to a solution of 7.52 mg/mL in 0.1 N NaOH and immediately brought to pH 7.2 by diluting to a concentration of 11.1 *μ*g/mL in PBS. The second group received phosphate buffered saline (PBS) in the same manner. At 4 weeks, the peak of hepatic infection (as shown in Figure 2), livers, and spleens were harvested for DNA and RNA extractions. PPAR and iNOS expression were determined by real-time RT-PCR and normalized to 18S RNA. Parasite load in the liver and spleen was determined by the real-time PCR procedure which was that of Nicolas and colleagues [45], except the reaction occurred in SYBR Green I PCR master mix (from Superarray). DNA, at 40 ng per reaction, was denatured at 95°C for 10 min, and then *Leishmania* kinetoplast DNA (kDNA) was amplified in a thermal cycler (Rotor-gene 6.0, from Corbett). The number of copies of kDNA per **μ**g of DNA was determined using a standard curve that was established with the cloned PCR product. Filled diamonds were curcumin-treated, and clear circles were saline controls. N indicates the number of mice in each group. Statistic analysis was performed by ANOVA after the data underwent natural log transformation as described in Adapala and Chan [16]. A *P* value of <0.05 was considered as significant.

PPAR is also regarded as dietary-sensing nuclear receptors; many activators of PPAR*γ* have been identified in foods [38]. Our laboratory is interested in the effect of curcumin (Figure 5), a dietary activator of PPAR*γ* on visceral leishmaniasis [16, 39]. It is the active principle in the spice turmeric, which is used abundantly in India, where visceral leishmaniasis is endemic in the Bihar region. Curcumin is well known for its anti-inflammatory effect, and there is ample evidence that the activity can be attributed to the activation of PPARs [40–43]. Zheng and Chen (2007) have suggested that there is a curcumin-responsive element residing in the regulatory region of the PPAR*γ* gene [44]. We examined the effect of curcumin on PPAR activation and *Leishmania* infection *in vivo* [16]. Susceptible BALB/c mice and resistant C3H mice were infected with *L. donovani*; immediately following inoculation, the mice were fed curcumin or phosphate-buffered saline (PBS) every other day. Then, at 4 weeks after infection, the livers and spleens were harvested and quantified for PPAR*γ*, iNOS, cytokines, and parasite load. Parasite load was quantified by two complementary methods, limiting dilution analysis and real-time PCR detection, and compared by the parametric test ANOVA after data transformation.  Figure 6 shows the results on PPAR*γ*, iNOS, and *Leishmania* kinetoplast DNA quantification. Curcumin treatment led to 5-fold increase in the gene expression for PPAR*γ* and 2-fold increase in the gene expression for PPAR*γ* in the spleen. It also caused an 80% decrease in the expression of iNOS in the liver and 68% in the spleen (Figures 6(a) and 6(b)). Concomitant with these modulations, parasite burden was elevated compared to the untreated vehicle control, (results from limiting dilution were not shown).

**Figure 7:**
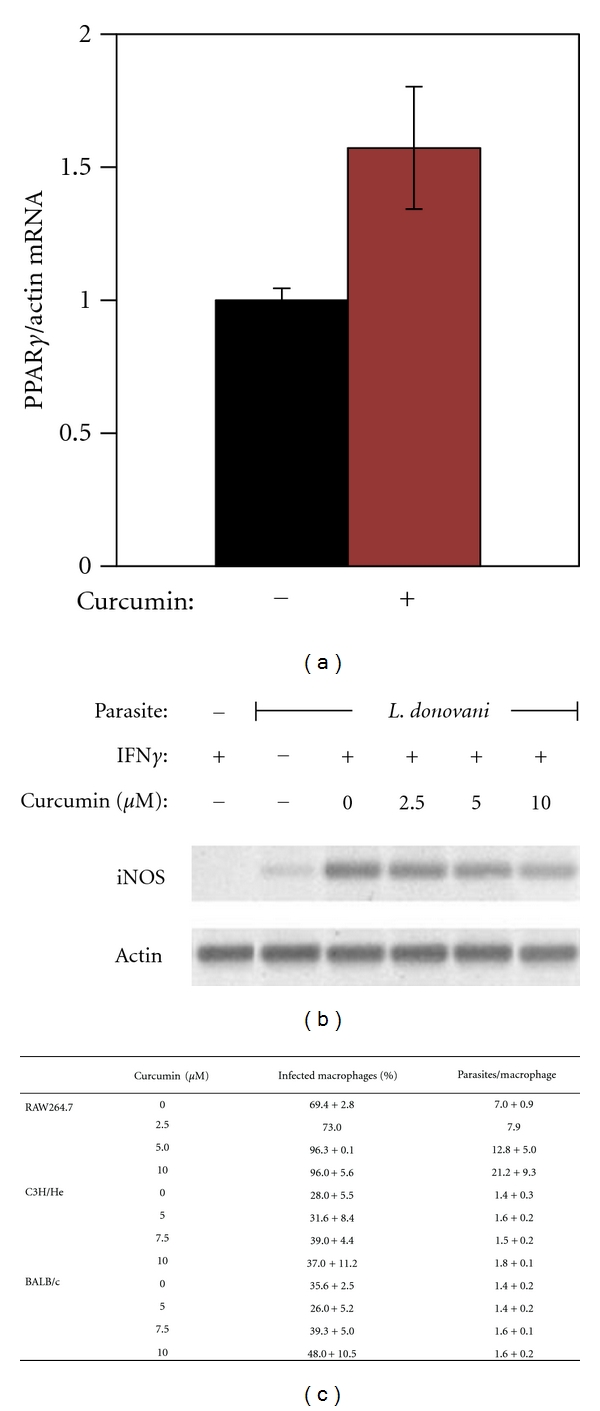
Curcumin induces PPAR*γ* mRNA expression and reduces iNOS mRNA expression in infected macrophages. In (a), PECs of BALB/c mice were infected with* L. donovani *promastigotes, and then 10 *μ*M of curcumin (brown bar) or vehicle control (0.1% acetone) was added. After 2 days, the cells were harvested for RNA isolation to determine the level of PPAR*γ* and *β*-actin expression by real-time RT-PCR. In (b), murine RAW264.7 cells were infected with *L. donovani *for 16–20 hours. Then, different concentrations of curcumin were added. Thirty minutes after curcumin treatment, IFN*γ* was added to activate the macrophages. At 5 hours after the addition of IFN*γ*, the cells were harvested, mRNA was extracted, and conventional RT-PCR was performed. The gel shows the end point PCR-amplified iNOS (496 bp) and *β*-actin cDNAs products. In (c), nonelicited PECs from resistant C3H and susceptible BALB/c strains were cultured with *L. donovani *promastigotes in wells that contained cover slips for a period of 16–20 hours; then curcumin and IFN*γ* and TNF*α* were added. On day 4-5, the coverslips were stained to enumerate the percent of infected macrophages and number of amastigotes per macrophage under a microscope, similar to steps described in Figure 3.

Corresponding to the feeding studies, we found that curcumin increased PPAR*γ* and decreased iNOS gene expression in infected macrophages. At 10 *μ*m, curcumin increased PPAR*γ* mRNA levels in infected peritoneal macrophages from BALB/c by 1.5-fold Figure 7(a). The dose dependency of the curcumin actions was demonstrated by iNOS gene expression and nitric oxide production. The level of gene expression is shown in Figure 7(b). At 10 *μ*m, curcumin reduced the level of steady-state RNA by 70%. The level of nitric oxide in the culture supernatants was also reduced. At 5, 7.5, and 10 *μ*m of curcumin, the reduction was 18, 39.3, and 61.4%, respectively [16]. In parallel to the reduction, parasite infectivity increased. The number of infected macrophages increased dose dependently from 28 to 37% in the resistant C3H strain and from 35 to 48% in the susceptible BALC/c strain. The number of amastigotes per macrophage also increased dose dependently, as shown in the table in Figure 7(c).

## 8. Conclusion: Mechanisms of PPARs on Leishmaniasis

**Figure 8:**
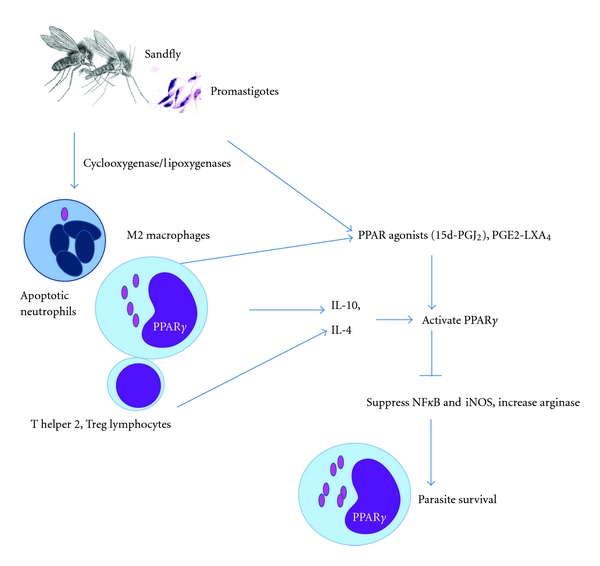
Scheme of *Leishmania* interaction with mediators of the resolution process during inflammation. When infected sandflies bite, promastigotes enter the neutrophils and macrophages that are recruited to the inflamed site of injection. The parasite can, by itself, activate the infected host cells to produce PPAR activators. This includes PPAR*γ* agonists such as the bioactive lipids 15d-PGJ_2_ and LXA_4_ from the arachidonic acid pathways. Engulfment of apoptotic neutrophils and IL-4 from T helper 2 cells can activate PPAR*γ* as well. Activation of PPAR*γ* polarizes the host macrophage towards the alternatively activated macrophage (M2) phenotype, which would produce arginase to divert substrate from iNOS and thus reduce the production of nitric oxide. As such, the parasite can survive and multiply within the host's macrophages, and the infection becomes chronic.

Taken together, these cumulative data from *L. donovani* and *L. major* infections indicate that PPAR plays a role in leishmaniasis, no matter in the liver where the PPAR*α* forms predominate, in the spleen and residential macrophages from the peritoneum or the bone marrow where the PPAR*γ* forms predominate [46]. Our perspective on how the nuclear factor is activated during infection and how its activation enhances the survival of *Leishmania* parasites is as follows. When the infected sandflies bite, an inflammatory reaction initiates innate and adaptive immune response for protection against the parasites. Neutrophils and macrophages are recruited to the injection site, and promastigotes enter the phagocytes. Launching a type 1 immune reaction, with production of nitric oxide, would resist infection. However, the parasites and infected host cells can synthesize ligands that activate PPAR*γ*. Phagocytosis of apoptotic neutrophils and IL-4 from T helper 2 cells can do so as well. With the activation of PPAR*γ*, *Leishmania *parasites would benefit from infiltration of macrophages, inactivation of the destructive inflammatory response, and promotion of the resolution of inflammation. Activation of PPAR promotes differentiation of the host macrophages into the alternatively activated (M2) macrophages, which have a type 2 phenotype and would produce arginase to interfere with enzymatic activity of iNOS [47]. As such, the parasites can survive and multiply within the host's macrophages, and the infection becomes chronic (Figure 8).

Currently, whether antagonists of PPAR would be therapeutic for leishmaniasis remains to be investigated. SR202, the antagonist that we used in our study, has been shown to prevent obesity in rats and therefore has *in vivo* efficacy. Ligands for PPAR*γ* are drugs for type 2 diabetes, and ligands for PPAR*α* are also currently in clinical use for obesity. How would these agents affect the outcome of leishmaniasis and whether PPAR affects the survival of other parasites are interesting questions [46].

## References

[1] Kedzierski L, Sakthianandeswaren A, Curtis JM, Andrews PC, Junk PC, Kedzierska K (2009). Leishmaniasis: current treatment and prospects for new drugs and vaccines. *Current Medicinal Chemistry*.

[2] Heinzel FP, Sadick MD, Holaday BJ, Coffman RL, Locksley RM (1989). Reciprocal expression of interferon *γ* or interleukin 4 during the resolution or progression of murine leishmaniasis. Evidence for expansion of distinct helper T cell subsets. *Journal of Experimental Medicine*.

[3] Nylén S, Gautam S (2010). Immunological perspectives of leishmaniasis. *Journal of Global Infectious Diseases*.

[4] Ji J, Masterson J, Sun J, Soong L (2005). CD4^+^CD25^+^ regulatory T cells restrain pathogenic responses during Leishmania amazonensis infection. *Journal of Immunology*.

[5] Chatelain R, Mauze S, Coffman RL (1999). Experimental Leishmania major infection in mice: role of IL-10. *Parasite Immunology*.

[6] Sacks D, Anderson C (2004). Re-examination of the immunosuppressive mechanisms mediating non-cure of Leishmania infection in mice. *Immunological Reviews*.

[7] Guimarães ET, Santos LA, Ribeiro dos Santos R, Teixeira MM, dos Santos WLC, Soares MBP (2006). Role of interleukin-4 and prostaglandin E2 in Leishmania amazonensis infection of BALB/c mice. *Microbes and Infection*.

[8] Chatelain R, Mauze S, Varkila K, Coffman RL (1999). Leishmania major infection in interleukin-4 and interferon-*γ* depleted mice. *Parasite Immunology*.

[9] Alexander J, McFarlane E (2008). Can type-1 responses against intracellular pathogens be T helper 2 cytokine dependent?. *Microbes and Infection*.

[10] Martinez FO, Helming L, Gordon S (2009). Alternative activation of macrophages: an immunologic functional perspective. *Annual Review of Immunology*.

[11] Munder M, Eichmann K, Modolell M (1998). Alternative metabolic states in murine macrophages reflected by the nitric oxide synthase/arginase balance: competitive regulation by CD4^+^ T cells correlates with Th1/Th2 phenotype. *Journal of Immunology*.

[12] Chawla A (2010). Control of macrophage activation and function by PPARs. *Circulation Research*.

[13] Gallardo-Soler A, Gómez-Nieto C, Campo ML (2008). Arginase I induction by modified lipoproteins in macrophages: a peroxisome proliferator-activated receptor-*γ*/*δ*-mediated effect that links lipid metabolism and immunity. *Molecular Endocrinology*.

[14] Rios FJO, Jancar S, Melo IB, Ketelhuth DFJ, Gidlund M (2008). Role of PPAR-gamma in the modulation of CD36 and FcgammaRII induced by LDL with low and high degrees of oxidation during the differentiation of the monocytic THP-1 cell line. *Cellular Physiology and Biochemistry*.

[15] Ricote M, Welch JS, Glass CK (2000). Regulation of macrophage gene expression by the peroxisome proliferator-activated receptor-*γ*. *Hormone Research*.

[16] Adapala N, Chan MM (2008). Long-term use of an antiinflammatory, curcumin, suppressed type 1 immunity and exacerbated visceral leishmaniasis in a chronic experimental model. *Laboratory Investigation*.

[17] Titus RG, Marchand M, Boon T, Louis JA (1985). A limiting dilution assay for quantifying Leishmania major in tissues of infected mice. *Parasite Immunology*.

[18] Reiner NE, Malemud CJ (1984). Arachidonic acid metabolism in murine leishmaniasis (Donovani): ex-vivo evidence for increased cyclooxygenase and 5-lipoxygenase activity in spleen cells. *Cellular Immunology*.

[19] Reiner NE, Malemud CJ (1985). Arachidonic acid metabolism by murine peritoneal macrophages infected with Leishmania donovani: in vitro evidence for parasite-induced alterations in cyclooxygenase and lipoxygenase pathways. *Journal of Immunology*.

[20] Lonardoni MVC, Barbieri CL, Russo M, Jancar S (1994). Modulation of Leishmania (L.) amazonensis growth in cultured mouse macrophages by prostaglandins and platelet activating factor. *Mediators of Inflammation*.

[21] Cuzzocrea S, Wayman NS, Mazzon E (2002). The cyclopentenone prostaglandin 15-deoxy-Δ^12,14^-prostaglandin J_2_ attenuates the development of acute and chronic inflammation. *Molecular Pharmacology*.

[22] Ribeiro-Gomes FL, Otero AC, Gomes NA (2004). Macrophage interactions with neutrophils regulate leishmania major infection. *Journal of Immunology*.

[23] Bonnans C, Fukunaga K, Levy MA, Levy BD (2006). Lipoxin A_4_ regulates bronchial epithelial cell responses to acid injury. *American Journal of Pathology*.

[24] Chan MMY, Moore AR (2010). Resolution of inflammation in murine autoimmune arthritis is disrupted by cyclooxygenase-2 inhibition and restored by prostaglandin E2-mediated lipoxin A4 production. *Journal of Immunology*.

[25] Bannenberg G, Serhan CN (2010). Specialized pro-resolving lipid mediators in the inflammatory response: an update. *Biochimica et Biophysica Acta*.

[26] Wenzel A, Van Zandbergen G (2009). Lipoxin A4 receptor dependent leishmania infection. *Autoimmunity*.

[27] El Kebir D, Filep JG (2010). Role of neutrophil apoptosis in the resolution of inflammation. *TheScientificWorldJournal*.

[28] Wanderley JLM, Moreira MEC, Benjamin A, Bonomo AC, Barcinski MA (2006). Mimicry of apoptotic cells by exposing phosphatidylserine participates in the establishment of amastigotes of Leishmania (L) amazonensis in mammalian hosts. *Journal of Immunology*.

[29] Savill J, Hogg N, Haslett C (1991). Macrophage vitronectin receptor, CD36, and thrombospondin cooperate in recognition of neutrophils undergoing programmed cell death. *Chest*.

[30] Van Zandbergen G, Klinger M, Mueller A (2004). Cutting edge: neutrophil granulocyte serves as a vector for Leishmania entry into macrophages. *Journal of Immunology*.

[31] Peters NC, Egen JG, Secundino N (2008). In vivo imaging reveals an essential role for neutrophils in leishmaniasis transmitted by sand flies. *Science*.

[32] Van Zandbergen G, Bollinger A, Wenzel A (2006). Leishmania disease development depends on the presence of apoptotic promastigotes in the virulent inoculum. *Proceedings of the National Academy of Sciences of the United States of America*.

[33] Noben-Trauth N, Kropf P, Müller I (1996). Susceptibility to Leishmania major infection in interleukin-4-deficient mice. *Science*.

[34] Doggrell S (2003). Do peroxisome proliferation receptor-*γ* antagonists have clinical potential as combined antiobesity and antidiabetic drugs?. *Expert Opinion on Investigational Drugs*.

[35] Liu J, Wu X, Mitchell B, Kintner C, Ding S, Schultz PG (2005). A small-molecule agonist of the Wnt signaling pathway. *Angewandte Chemie*.

[36] Soller M, Tautenhahn A, Brüne B (2006). Peroxisome proliferator-activated receptor *γ* contributes to T lymphocyte apoptosis during sepsis. *Journal of Leukocyte Biology*.

[37] Odegaard JI, Ricardo-Gonzalez RR, Goforth MH (2007). Macrophage-specific PPAR*γ* controls alternative activation and improves insulin resistance. *Nature*.

[38] Martin H (2010). Role of PPAR-gamma in inflammation. Prospects for therapeutic intervention by food components. *Mutation Research*.

[39] Chan MMY, Adapala NS, Fong D (2005). Curcumin overcomes the inhibitory effect of nitric oxide on Leishmania. *Parasitology Research*.

[40] Chan MMY, Huang HI, Fenton MR, Fong D (1998). In vivo inhibition of nitric oxide synthase gene expression by curcumin, a cancer preventive natural product with anti-inflammatory properties. *Biochemical Pharmacology*.

[41] Jacob A, Wu R, Zhou M, Wang P (2007). Mechanism of the anti-inflammatory effect of curcumin: PPAR-*γ* activation. *PPAR Research*.

[42] Kang Q, Chen A (2009). Curcumin suppresses expression of low-density lipoprotein (LDL) receptor, leading to the inhibition of LDL-induced activation of hepatic stellate cells. *British Journal of Pharmacology*.

[43] Lin J, Chen A (2008). Activation of peroxisome proliferator-activated receptor-*γ* by curcumin blocks the signaling pathways for PDGF and EGF in hepatic stellate cells. *Laboratory Investigation*.

[44] Zheng S, Chen A (2007). Disruption of transforming growth factor-*β* signaling by curcumin induces gene expression of peroxisome proliferator-activated receptor-*γ* in rat hepatic stellate cells. *American Journal of Physiology*.

[45] Nicolas L, Prina E, Lang T, Milon G (2002). Real-time PCR for detection and quantitation of Leishmania in mouse tissues. *Journal of Clinical Microbiology*.

[46] Chan MM, Evans KW, Moore AR, Fong D (2010). Peroxisome proliferator-activated receptor (PPAR): balance for survival in parasitic infections. *Journal of biomedicine &amp; biotechnology*.

[47] Chawla A (2010). Control of macrophage activation and function by PPARs. *Circulation Research*.

